# Sleep and its relationship to health in parents of preterm infants: a scoping review

**DOI:** 10.1186/s12887-018-1320-7

**Published:** 2018-11-12

**Authors:** Gunhild Nordbø Marthinsen, Sølvi Helseth, Liv Fegran

**Affiliations:** 1 Department of Health and Nursing Sciences, Faculty of Health and Sports Science, University of Agder, 4604 Kristiansand, Norway; 2 Department of Nursing and Health Promotion, Faculty of Health Sciences, OsloMet- Oslo Metropolitan University, NO-0130 Oslo, Norway

**Keywords:** Scoping review, Sleep, Health, Parents, Mother, Father, Preterm, Nursing

## Abstract

**Background:**

Sleep is essential for human health and functioning. Parents of preterm infants are susceptible to sleep disturbances because of stress related to the preterm birth. Poor sleep has the potential to affect parental health and well-being. The aim of this study was to identify and map evidence on sleep and its relationship to health in parents of preterm infants. No review has summarized the evidence on this topic.

**Methods:**

A scoping review was conducted. Seven health and medical electronic research databases were searched for relevant quantitative and qualitative primary studies, including grey literature. The search was performed March 2–7, 2017.

**Results:**

Ten American studies and one Australian study were included in the review. Most research was quantitative and focused on maternal sleep and mental health within the first two weeks after the childbirth. Both objective and subjective sleep measures were used to study sleep at the hospital; actigraphs were not used after discharge. Maternal sleep was poor early postpartum, and this was associated with negative health outcomes. Two cohort studies compared sleep in mothers of preterm and term infants, but the results were conflicting. In one qualitative study, fathers described their inability to catch up on sleep after homecoming with a preterm baby.

**Conclusions:**

Quantitative studies reporting on maternal sleep early postpartum was most frequently occurring in the results. Qualitative research on the topic was identified as a knowledge gap. More cultural and geographical breadth, including research on fathers’ sleep, is recommended in future research.

## Background

Every year, approximately 15 million infants around the world are born before 37 completed weeks of gestation, and the rate of preterm birth is increasing [1]. Globally, preterm birth is the second highest direct cause of death in children younger than 5 years [2]. Depending on the degree of prematurity and severity of disease, the preterm infant requires hospitalization and technological care in a neonatal intensive care unit (NICU) [3]. The event of a preterm birth has been associated with maternal and parental distress [4–8]. Recent studies have reported negative effects on parental sleep because of feelings arising from the preterm birth experience [9, 10]. Sleep is important for parents’ own physical and emotional health, as well as for their abilities to cope with illness, support their child and family members, participate in decision making and maintain relationships [11]. Sleep is also a critical determinant of physical and mental health [12]. Parents may benefit from postpartum nursing care that prioritizes sleep given that parents are experiencing a critical time for healing [13]. This literature review was a scoping review of the existing evidence on sleep and its relationship to health in parents of preterm infants. The findings are relevant to healthcare providers in NICUs. An understanding of parental sleep after the incidence of preterm birth might be the first step toward developing strategies and interventions to promote sleep and health in this parent population.

Sleep is a multidimensional, biobehavioural process that is essential for human health and functioning [14]. Although the functions and mechanisms of sleep are not yet fully understood, it is generally accepted that sleep entails restorative mechanisms and aids in the physiological and emotional regeneration of individuals [15, 16]. Sleep performs essential functions in restoring human energy, conserves energy and body metabolism, keeps physiological systems within proper homeostatic limits, maintains host defences, and restores physiological processes that have progressively degraded during wakefulness [16]. Sleep deprivation has been associated with deficits in function across a wide range of indicators of psychological, interpersonal, and somatic well-being [17]. Increasing evidence points to a bidirectional relationship between sleep and health; sleep disturbances contribute to the development of or increase the severity of various medical and psychiatric disorders. Such disorders also have a negative impact on sleep [18]. It is generally accepted that 7–8 h is the optimal amount of sleep needed per night for adequate daytime functioning and to reduce the risk of developing serious medical conditions [12].

During the postpartum period, sleep disturbances are common among new parents [19]. The postpartum (or postnatal) period begins immediately after birth; the initial or acute postpartum phase, refers to the first 6–12 h after childbirth, the subacute postpartum period, refers to 2–6 weeks after birth, and the delayed postpartum period refers to the period up to 6 months after birth [20]. In the subacute and delayed postpartum period, parents of preterm infants are reported to be susceptible to poor sleep because of stress [9, 10]. Parents are often subject to psychological distress related to the infant’s health, treatment, survival and risk of disability [21]; the use of complex medical language and technology in the NICU [22]; and the loss of the parental role [21–23]. The emotional burden on parents can last for months [7, 24]; mothers have continued to report high levels of emotional stress [5, 7], and depression [5, 25] after their discharge from hospital. Prematurely born children are also likely to have more sleep problems than full term infants [26], and the sleep problems may last throughout the early years [27]. The role of sleep and its impact on health outcomes for these parents seems to be complex.

In healthy postpartum women, poor sleep has been associated with stress and adverse wellbeing [28], fatigue [29], and depression [10, 30, 31]. Poor parental sleep can negatively affect the parent-child relationship [32] and have a negative effect on parent and family relationships [33]. The mental health of parents with hospitalized neonates has been an increasing concern for clinical paediatric workers in recent years [34]. Parental mental health and parents’ ability to be responsive and sensitive to the needs of the preterm infant have been found to be crucial factors in the long-term development of very preterm infants [35, 36]. Establishment of this early physical and emotional contact is important for both the infant and the parents [37]. Sleep-disrupted parents may have fewer opportunities for this important early contact with their child; parents have described negative effects on daily functioning, well-being, and parenting as a result of fatigue caused by sleep disruption [38]. Thus, adequate sleep for parents is crucial to their psychological functioning and ability to support and participate in care for their child [39]. To our knowledge, no review has summarized the existing knowledge of the sleep and health characteristics of parents of preterm infants in the NICU and studied the relationships between sleep and health in this population over time. Therefore, there was a need to summarize the existing evidence on this topic.

## Methods

The objective of this scoping review was to identify and map information on sleep and its potential relationships to parental health among parents of preterm infants. More specifically, the review focused on the following questions:

1. What study designs have been used to investigate relationships between sleep and health in parents of preterm infants?
2. Which research instruments have been used to study relationships between sleep and health in parents of preterm infants?
3. What outcomes have been reported regarding sleep and its relation to health in parents of preterm infants?

This scoping review was based on the methodology and guidance for conducting systematic scoping reviews developed by Arksey and O’Malley [40] and further expanded by Levac and colleagues [41]. Levac et al.’s recommendations for refining the methodology included to clearly articulate the research question and link the aim and research questions (stage one); combine feasibility with range and extensiveness of the scoping process (stage two); using an iterative team- based approach in the study selection process (stage three); extracting data (stage four); integrating a numeric summary and qualitative thematic analysis, reporting outcomes, and considering the consequences of study results for policy practice or research (stage five); and finally, incorporating discussion with stakeholders as a compulsory knowledge translation part of the scoping process (stage six) [41]. In this review, no consultation with stakeholders was performed. According to the recommendations, a suitable team, with content and methodological expertise, was established early in the process to ensure a successful completion of the review. The results are presented as a descriptive numerical summary and textually.

### Search terms and search strategies

**Fig. 1:**
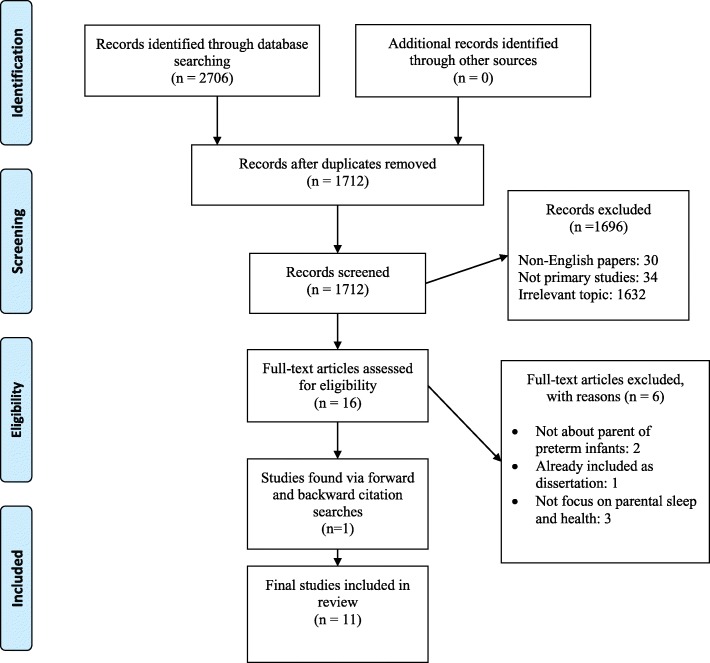
PRISMA flow Diagram for the scoping review

The search strategy aimed to trace both published and unpublished studies up to March 7, 2017. To prepare for the search process, an identification of the main concepts inherent in the research questions was guided by the elements of a PICOC structure (population, intervention/exposure, comparison, outcome, context) [42]. Three main concepts were identified for the development of search strategies. These concepts were population: parents of preterm infants; interest: sleep; and context: hospital or home settings. A three-step search strategy was performed. First, an initial limited search in Ovid Medline and Cinahl plus with full text (EBSCOhost) was undertaken, followed by an analysis of the text words contained in titles and abstracts, as well as an analysis of the index terms used to describe each article. A second search, using all identified keywords and index terms, was modified and adapted to each database: CINAHL Plus with Full Text (EBSCOhost), MEDLINE, Embase, PsycINFO (all via Ovid SP), Proquest, and Web of Science. The searches were performed based on a building block search strategy [43]. Each main word from PICO was represented by a block of keywords / single words / phrases or controlled nouns. Individual search terms in the same block were combined with OR. Each block was searched separately, and finally, the search boxes were combined with AND so that at least one word from each search block was to be included in the final search block. The proximity operator was used to ensure that words for sleep and parents would appear close to each other (Cinahl; N8 and Medline; adj 9). Truncation marks* were used to search word trunks. In CINAHL Plus with Full Text (EBSCOhost), the key search words included (Mesh headings) (“Parents” +) OR (maternal* OR paternal* OR parent* OR mother* OR father*) OR (Sleep +) OR (“Sleep disorders +”) OR (“Wakefulness”) OR sleep* OR (Infant, Premature) OR (“Infant, Low Birth Weight +”) OR (“Childbirth, Premature”) OR (“Intensive Care Units Neonatal”). Keywords used were neonat* OR NICU OR prematur* OR preterm* OR birth weight OR (sleep OR insomnia OR awake OR asleep OR wake OR wakeful* OR REM) N8 (parent* OR mother* OR father* OR caregiver* OR maternal*). After identifying studies, the reference lists of all included studies were searched for additional literature. Citation searches included searches in Google scholar, Scopus, Ovid SP, PubMed and Web of Science. The search for unpublished studies included Prospero and Proquest. The searches were conducted March 2–7, 2017. Figure 1 presents a PRISMA flow diagram from search to the final inclusion of the studies according to Moher et al. [44] (Figure 1).

### Search outcome

After the searches in seven electronic databases, the identified papers were transferred to Endnote Reference Manager for removal of duplicates and further exported into a Microsoft Excel format for screening of titles and abstracts. Only studies meeting the inclusion and exclusion criteria were eligible for inclusion in the review. The inclusion criteria were the following: primary studies of quantitative or qualitative design published in English, reporting on sleep in parents (mothers or fathers) of preterm infants (infants born before gestational week 37), and parents’ health issues up to one year after the birth of the preterm infant. Health aspects were understood according to the World health organization (WHO’s) definition of health [45] and categorized as health concerns about social, physical, or psychological well-being. The exclusion criteria used in the review were the following: not primary studies, studies published in languages other than English, and studies not reporting on parental sleep and health. According to the requirements of the screening process, the team met to discuss decisions surrounding the inclusion and exclusion of studies. Studies were screened independently by two reviewers, and any disagreements were resolved during the screening process. Reviewers met at the beginning, midpoint and final stages to discuss challenges and uncertainties related to study selection, as recommended by Levac et al. [41].

### Quality appraisal

Based on the current methodological guidelines for scoping studies [40, 41], no critical appraisal of the strength and quality of the included papers was performed.

### Data abstraction

A data extraction sheet was developed to determine which variables to extract to answer the research questions. Each study was screened and extracted according to author, year of publication, country of origin, study design, purpose of study, population, and research instruments used to study sleep and health. A summary of the key findings was also extracted from each study. The results are presented in tables (Tables 1 and 2). Data were extracted by one reviewer and discussed with two other reviewers. Uncertainties and disagreements resulting from data abstraction were resolved through discussion.

**Table 1:** Key information included studies

Author, Country	Purpose	Study design	Sample	Key findings
Lee & Kimble (2009), USA	Explore relationships between impaired sleep and wellbeing in mothers with low birth weight infants (LBW) in the NICU.	Cross sectional	20 mothers of preterm LBW infants in the NICU	Poor sleep quality and disturbed daytime function, night-time TST was ≤7 h. The mothers took more time to fall asleep compared to normal adults. Daytime sleep was < 1 h. Mothers reported moderate depressive symptoms. HRQoL was 1 SD below the normative score for age-matched females in the US. Mothers with more sleep debt reported more fatigue severity, depression and poorer HRQoL.
Lee & Hsu (2012a), USA	Examine the relationship among sleep, stress, depression, fatigue and H- QoL among mothers with a LBW infant in the NICU early postpartum.	Cross sectional	55 mothers of preterm LBW infants in the NICU	Poor sleep quality in mothers was associated with stress, fatigue, depression and poor HRQoL. Maternal stress contributed to poor sleep quality and depression, which in turn contributed to poor HRQoL.
Lee et al. (2012b), USA	Describe daytime activity levels and their associations with sleep, fatigue, depressive symptoms and quality of life.	Cross sectional	51 mothers of preterm infants in the NICU	Compared to high activity mothers, mothers with low activity levels slept less at night-time and napped more during the daytime, and they reported more postpartum depressive symptoms. Higher daytime activity was associated with fewer depressive symptoms. More sleep was associated with less severe fatigue.
Lee & Hsu (2016), USA	Examine whether depressive symptoms and sleep disturbance in black mothers would vary as a function of the 5-HTTLPR when they faced the stress of infant hospitalization after preterm birth early postpartum.	Cross sectional	30 mothers of preterm LBW infants in the NICU	Mothers with the L/L allele reported greater sleep disturbances than those with the S/L allele. Mothers’ perceived global stress, depressive symptoms, and circadian activity rhythms did not vary with their 5- HTTLPR genotype.
Shelton et al. (2014), USA	Compare the levels of self-reported perceived global and situational stress, sleep disturbance and the level of wellness between mothers with an infant in the NICU who are categorized as having high or low depressive symptoms.	Cross sectional	55 mothers of preterm infants in the NICU	All the mothers in this study experienced poor sleep. Mothers reported a moderate level of morning fatigue, and their HRQoL for physical and mental components were below the norm. Mothers with higher depressive symptoms reported greater stress and experienced poorer sleep.
Schaffer (2012), USA	Describe maternal and infant factors that influence sleep quality; examine the relationships between depression, anxiety, stress, social support and sleep quality, and describe the influence of a RGI intervention on sleep quality among a sample of mothers whose preterm babies were admitted to the NICU.	Prospective descriptive data analysis. Clinical trial.	20 mothers of preterm infants in the NICU	Anxiety, depression, stress and lower income were related to poor sleep quality; social support and increased age were related to better sleep quality. With cumulative R-GI use, sleep quality improved. The participants reported that the intervention of R-GI assisted them in falling asleep and in reducing stress.
Lee et al. (2013), USA	Examine the effectiveness of a 3-week bright light therapy RCT intervention on sleep and health outcomes of mothers with LBW infants in the NICU.	Clinical trial	30 mothers of preterm LBW in the NICU	Mothers in the treatment group improved in nocturnal TST, CAR, morning fatigue, depressive symptoms, and HRQoL compared to the control group. The 3-week bright light intervention combined with sleep hygiene materials appeared promising for maternal sleep early postpartum.
Williams & Williams (1997), USA	To assess simultaneous interactions among variables in a path diagram. The variables assessed were caregiver fatigue, sleep effectiveness, perception of stress, reframing capacity within the family, family cohesion, family income, and the placement of a preterm on an apnea monitor.	Cohort	74 mothers of preterm infants in the NICU and at home	Path diagrams increased in complexity over time. At all measure points, sleep effectiveness tended to decrease fatigue. When sleep effectiveness increased, the levels of fatigue decreased.
Gennaro & Fehder (2000), USA	Examine the difference in health behaviours among mothers of preterm, VLBW infants and mothers of healthy term infants.	Cohort	64 mothers of VLBW preterm infants and 60 mothers of full-term infants in the NICU and at home	No differences were noted in sleep between mothers of preterm infants and term infants. The amount of sleep per night did not change significantly over time; the mothers were successful in managing sleep.
McMillen et al. (1993), Australia	Compare the effects of the demands of term and preterm infants on the daily rhythms of sleep and wakefulness and salivary melatonin and cortisol concentrations in mothers.	Cohort	23 mothers of term infants and 22 mothers of preterm infants at home.	Mothers of preterm infants slept less, were more awake, had less time asleep and fewer sleep bouts per 24 h compared to the mothers of full-term infants. Cortisol and melatonin salivary tests varied between the groups, maybe because of greater physiological disruption in mothers of preterm infants.
Wollenhaupt (2010), USA	Explore the experience of mothers and fathers as they integrate their premature infant into the family at home.	Naturalistic inquiry	Parents of 10 preterm infants at home	After coming home with the baby, most of the parents described their sleeping experiences like soldiers in combat. Parents had a heightened awareness of sounds in the night, stood guard over and wakened to check on the baby. The best nights of sleep consisted of 3–5 h of interrupted sleep. Fathers described their inability to catch up on sleep; they went to work early or awakened to take care of the baby, so the mother could sleep; and they had less opportunity to take naps during the daytime compared to mothers.

Abbreviations Table 1: *TST* total sleep time, *VLBW* very low birth weight, *NICU* neonatal intensive care unit, *LBW* low birth weight, *RCT* randomized control trial, *R-GI* relaxation guided imagery intervention, *HRQoL* health related quality of life, *SD* standard deviation, *CAR* circadian activity rhythm, *5-HTTLPR* Serotonin-transporter-linked polymorphic region

**Table 2:** Study designs and instruments used to study sleep and health

Author Year Country	Design	Sample	Context	Time data collection	Instruments used to study sleep	Instruments used to study health
Lee & Kimble (2009), USA	Quantitative, cross sectional	20 mothers of LBW infants	NICU	Second week postpartum	Self-report: Sleep rated for the past week (GSDS), sleep diary (2 days) Objective: actigraphy for 2 days.	Fatigue: NRS-F Depression: EPDS Health related quality of life: SF36v2
Lee & Hsu (2012a), USA	Quantitative, cross sectional	55 mothers of LBW infants	NICU	Second week postpartum	Self-report: Sleep rated for the past week (GSDS), SDI, sleep diary. Objective: Actigraphy 2 days (*N* = 20) and 3 days (*N* = 35)	Fatigue: LFS Depression: EPDS Stress: PSS and IES Health related quality of life: SF36v2
Lee et al. (2012b), USA	Quantitative, cross sectional, comparative	51 mothers of preterm infants	NICU	Second week postpartum	Self-report: Sleep rated for the past week (GSDS), sleep diary. Objective: Actigraphy 2–3 days	Fatigue: LFS Depression: EPDS Health related quality of life: SF36v2 Activity: actigraphy to measure rest/activity pattern
Lee & Hsu (2016), USA	Quantitative, cross sectional, comparative	30 mothers of LBW infants	NICU	Second week postpartum	Subjective: Sleep rated for the past week (GSDS), sleep diary. Objective: Actigraphy 3 days	Depression: EPDS Stress: PSS Serum: Test serotonin transporter polymorphism (5 HTTLPR) genotype.
Shelton et al. (2014), USA	Quantitative, cross sectional comparative design	55 mothers of preterm infants	NICU	Second week postpartum	Self-report: Sleep rated for the past week (GSDS), sleep diary (2–3 days). Objective: Actigraphy 2–3 days	Fatigue: LFS Depression: EPDS Stress: PSS Health related quality of life: SF36v2
Schaffer (2012), USA	Quantitative Prospective descriptive data analysis Clinical trial	20 mothers of preterm infants	NICU	Repeated measures over eight weeks postpartum	Self-report: PSQI	Anxiety: STAI Depression: CES-D Stress: PSS Social support: FSSQ + A brief semi structured interview on the acceptability of the R-GI intervention.
Lee et al. (2013), USA	Quantitative, clinical trial	30 mothers of LBW infants randomized to a treatment or control group.	NICU	Data collected at pretreatment (second week postpartum) and after 3-week intervention.	Self-report: Sleep rated for the past week (GSDS), sleep diary for 3 days Objective: Actigraphy 3 days	Fatigue: LFS Depression: EPDS Stress: PSS Health related quality of life: SF36v2 Maternal perceived support: FSS
Williams & Williams (1997), USA	Quantitative, cohort, comparative	74 mothers of preterm infants	NICU and home T1/Baseline = NICU T2 = one-week post-discharge T3 = one-month post-discharge.	Data were collected at three-time periods; baseline, one-week post discharge and one-month post discharge	Self-report: Subscale of the VHS Sleep Scale.	Stress: PSS Fatigue: MAF, Reframe: FCOPES, Cohesion: FACES II
Gennaro & Fehder (2000), USA	Quantitative, longitudinal, comparative	124 mothers, 64 with a VLBW preterm infant and 60 with a full-term infant	NICU and home	Data collected within 24 h after birth, + home (1 month, 2 months, and 4 months postpartum)	Self-report: SWAI and SSS.	Maternal weight loss, Nutritional intake: 24-h diet recall, Exercise: FWPA
McMillen et al. (1993), Australia	Quantitative, cohort, comparative	Mothers of 23 term infants and 22 preterm infants	Home	Up to 5 months after either birth (term group) or arrival of the infant home (preterm group).	Self-report: 24-h sleep/wake chart for infant and mother (completed by mother).	Saliva: Melatonin and cortisol tests.
Wollenhaupt (2010), USA	Qualitative, Natural inqury design	Mothers and fathers in 10 families.	Home	3–5 weeks following discharge from NICU	Semi-structured interviews	

Abbreviations: *SDI* Sleep deviation Index, *LFS* Lee Fatigue Scale, *PSS* Perceived Stress Scale, *MAF* Multidimensional Assessment of Fatigue, *FCOPES* subscale of the Family Crisis Oriented Personal Evaluation Scale, *FACES II* Cohesion subscale of the Family Adaptability and Cohesion Scale, *FWPA* the four-week physical activity questionnaire, *SF36v2* The Medical Outcomes Short Form 36 version 2, *FSS* Family Support scale, *EPDS* Edinburgh Postpartum Depression scale, *FSSQ* The Duke University of North Carolina Functional Social Support Questionnaire, *STAI* State-Trait Anxiety Inventory, *CES-D* Center for Epidemiological Studies Depression Scale, *SWAI* Sleep Wake Activity Inventory scale, *SSS* Stanford Sleepiness Scale 52, *GSDS* General Sleep Disturbance scale, *VHS sleep scale* Verran and Snyder-Halpern Sleep Scale (VSH), *PSQI* The Pittsburgh Sleep Quality Index, *NICU* neonatal intensive care unit, *LBW* low birth weight, *VLBW* very low birth weight

### Synthesis

Consistent with the methodology used in this review, the collating, summarizing and reporting of results were guided by Levac et al.’s recommendations [41]. The methodological process of synthesis was performed in three distinct steps. First, analyses of the included data were performed. Secondly, the results are presented in tabular form (Tables 1 and 2). Thirdly, meaning was applied to the results. Through repeated readings of each article, a thematic analysis was performed according to the purpose and research questions of the review, a process similar to the analytical technique used for qualitative data [41]. The meaning of the findings as they related to the purpose and research questions were discussed with cooperating authors. The researchers decided that the best approach to stating the outcomes and findings was a combination of results presented in tabular form (Tables 1, 2 and 3), followed by a narrative, analytical text responding to each research question.

**Table 3:** Significant correlations identified between sleep and health

Author, year of publication, country of origin	N=	Sleep	Mental health					Wellbeing		Social health
			Fatigue		Anxiety	Stress	Depression	HRQOL		Social support
			Morning	Evening				Mental	Physical	
Lee & Kimble (2009), USA	20	GSDS	.52*	.51*	–	–	ns	−.53*	ns	
		Sleep quality	ns	.54*	–	–	ns	−.55*	ns	
		Daytime function	.55*	.52*	–	–	ns	−.48*	−.45*	
		TST	ns		–	–	ns	ns	ns	
		WASO			–	–		ns	ns	
		Sleep debt	.48*	ns	–	–	ns	ns	ns	
Lee et al. (2012b), USA	51	CAR	ns		–	–	ns	ns	ns	
		TST	−.30*	–	–	–	ns	ns	ns	
		WASO	ns		–	–	ns	ns	ns	
		Sleep quality	.38**	–	–	–	ns	−.49*	−.38*	
Lee & Hsu (2012a), USA	55	Sleep quality index	.54**		–	.36**	ns	−.49*	−.34*	
		TST	ns		–	–	–	ns	ns	
		WASO	ns		–	–	–	ns	ns	
		SDI	ns		–	–	–	ns	ns	
Schaffer (2012), USA	20	Sleep quality	–		.514*‡	ns	.496*†	–		−.462* †
Gennaro & Fehder (2000), USA	23	SWAI	ns							
		SSS								

Notes: Asterisk (*) indicated correlation was significant at *p* < .05, (**) indicated correlation was significant at *p* < .01. ns = not statistically significant, − not measured, † baseline value, ‡ Time 2 measure point value

Abbreviations: *GSD*S General Sleep Disturbance scale, *WASO* wake after sleep onset in minutes, *CAR*, circadian activity rhythms, *SDI* sleep deviation index, *TST* nighttime total sleep time monitored from actigraphs, *SWAI* the sleep wake activity inventory, *SSS* the Stanford Sleepiness Scale

## Results

After completing the screening process, eleven studies were ultimately included in the review. Nine studies were retrieved as articles [46–54], and two were retrieved as dissertations [55, 56] (Table 1). Schaffer had published a paper based on a dissertation [56, 57], but because the dissertation contained more data, only it was included in the review. The amount of research on the topic was found to increase over time, with eight studies published after 2009, and the majority of the literature was geographically concentrated in the United states (U.S.) (Table 1). The existing evidence was dominated by quantitative literature concerning maternal sleep and mental health in the early postpartum phase. Only three studies were concerned with maternal sleep and health characteristics over time after discharge from the NICU wards (Table 1). Fathers were only represented in one qualitative study. Table 1 gives an overview of the key information of the included studies.

### Study designs used to investigate sleep and health in parents of preterm infants

Ten of the included studies used a quantitative design, and only one used a qualitative design (Table 1). Among the quantitative literature, five studies used a cross sectional design [47–51], and three were cohort studies [52–54]. Schaffer used a prospective, repeated data analysis performed over an 8-week period [56]. Two clinical trials were identified [46, 56]. One qualitative study was based on a naturalistic inquiry design, as described by Lincoln and Guba [55]. Table 1 presents an overview of the study designs.

### Questionnaires, diaries and actigraphs used to study sleep in parents of preterm infants

In the quantitative studies, a variety of research instruments were used to study sleep. Six papers reported on objective sleep data derived from wrist actigraphs, which are small monitors used to assess rest-activity patterns [58]. The actigraphs were used to study maternal sleep early postpartum for short periods of 2–3 days, and they were not used in research performed after discharge from hospital (Table 2). The objective sleep measures from actigraphs were supplemented with patient-reported outcome measures (PROMs), standardized measures developed to capture patient-reported outcomes [59]. Sleep questionnaires and sleep diaries were examples of PROMs used to assess sleep. Sleep diaries were subjective sleep assessments of individuals’ sleeping and waking times, accompanied by related information [60]. The sleep questionnaires were used to evaluate various aspects of sleep, such as sleep disturbances, sleep quality, sleep characteristics and sleepiness (Table 2). The spectrum and breadth of research tools used to assess sleep in the early postpartum period provided very detailed knowledge of sleep characteristics during this phase. The breadth and specificity of research tools used after discharge from hospital was not so distinct; PROMs were used to evaluate (daytime) sleepiness [53] and maternal sleep characteristics [52]. Compared to the early postpartum period, the insights derived from these tools were less specific. The instruments used to study sleep are presented in Table 2.

### Questionnaires and research instruments used to study health in parents of preterm infants

Different research tools have been used to study health, with PROMs being used most commonly in the literature. Only one study reported on objective measures exclusively [54]. Twelve different PROMs were identified, with assessments of depressive symptoms, anxiety, stress, fatigue, health-related quality of life (HRQoL), social support, reframe, cohesion, and physical health (Table 2). Physical health was studied with objective measures such as actigraphs to explore daily rest/activity patterns, measures of body weight, and blood samples (Table 2). In the research, the most frequently studied aspect of health was mental health, followed by HRQoL and physical health. Social health was the least studied health component. Table 2 provides an overview of the research instruments used to study health.

### Sleep and health in parents of preterm infants

In the early phase after childbirth, maternal sleep was described as poor. Data derived from actigraphs indicated that maternal total sleep time (TST) was less than 7 h [47–51]. Sleep was also fragmented, with frequent nightly awakenings and increased sleep time during the daytime [47, 49, 50]. According to PROMs, the mothers evaluated their sleep as poor [47, 48, 56]. Surprisingly, many of the mothers slept poorly despite the fact that they spent their nights at home and did not provide care for their hospitalized preterm infants [47–51]. The post-discharge experiences of mothers with preterm infants were described as complex [52]. Two cohort studies compared sleep between mothers of preterm and term infants over time [53, 54] and found contradictory results. Gennaro and Fehder [53] did not find any differences in sleep between the two groups of mothers at any measure points, while McMillen et al. [54] found that mothers of preterm infants slept less and had fewer sleep bouts compared to mothers of term infants. The findings did not clearly suggest that the mothers of preterm infants experienced poorer sleep compared to mothers of term infants over time. Additionally, qualitative literature supported the notion that sleep was challenged after coming home with the preterm infant [55]. The majority of parents described their sleeping experiences in a similar fashion to soldiers in combat. Parents described how they got small bursts of sleep in a variety of diverse ways; some parents tried to catch sleep whenever they could because they were so affected by lack of sleep [55]. In this study, the fathers described their inability to catch up on sleep. They took care of the baby so the mother could sleep, or they went to work early in the morning and had no opportunity to nap during the day compared to the mothers [55].

According to the outcomes of their sleep, some mothers were more susceptible than others to experiencing poor sleep. Shelton et al. [51] compared mothers of preterm infants with high or low depressive symptoms and reported poorer sleep in the group with high depressive symptoms. Lee et al. [47] compared mothers with high and low daytime activity levels and reported poorer sleep in the group with low activity levels. Additionally, Lee and Hsu [50] examined whether depressive symptoms and sleep disturbances would vary as a function of the 5-HTTLPR genotype (the short allele of 5-HTTLPR has been associated with depression and sleep disturbances). Surprisingly, the mothers with the long allele for the genotype reported greater sleep disturbances compared to mothers with the short (S/L) allele.

### Interventions to promote sleep and health in parents of preterm infants

Two clinical trials were identified in the material. Both interventions were developed to promote maternal sleep and health early postpartum. Lee et al. [46] tested the effect of bright light therapy on sleep and health outcomes in mothers of preterm infants hospitalized in the NICU. The intervention was evaluated as feasible, since the mothers in the treatment group showed improvements in TST and self-rated sleep quality. Schaffer [56] tested the effects of an eight-week relaxation guided imagery intervention (R-GI) on maternal sleep quality. The R-GI intervention was found to be an effective strategy to improve maternal stress and coping early postpartum in the NICU.

### Associations between sleep and health in parents of preterm infants

As shown above, different health outcomes were reported in the included studies (Table 1). Only five studies computed data analysis of statistical correlations among sleep and health variables [47–49, 53, 56], and four of these found statistically significant results (Table 3). The qualitative study also presented findings suggesting an association between sleep and health [55]. In the quantitative studies, a positive significant correlation was reported between sleep and maternal fatigue [47–49], sleep and anxiety [56], sleep and stress [48], and sleep and depression [56]. The positive correlation showed that high scores for poor sleep were associated with high scores for stress, fatigue and anxiety in mothers early postpartum (Table 3). Social support was negatively correlated with sleep [56]; when social support increased, quality-of-sleep scores decreased (decreased sleep quality scores indicated better sleep quality). Additionally, lower HRQoL (mental and physical) was associated with poorer levels of sleep quality [47, 49] and daytime functioning [49]. The qualitative literature suggested associations between sleep and parental health [55]. In Wollenhaupts dissertation [55], parents expressed how sleep loss after homecoming with a preterm baby affected their daily life. The parents felt exhausted and run down, had less ability to think clearly, and cope with daily life situations. Lack of sleep also impacted the parents’ relationship in a negative way. Getting from 2 to 5 h of sleep at night over a period of weeks lead to such feelings [55]. Table 3 presents an overview of the significant correlation identified between sleep and health.

## Discussion

In neonatal care, care provision is influenced by parental mental health and well-being [34]. The ability of parents to establish emotional closeness to the preterm infant may be crucial to the wellbeing of the infant [61] and has been shown to have a long-term impact on the function of affective relationships and healthy development outcomes [62]. An understanding of parents’ sleep and health characteristics in the postpartum period can contribute valuable insights into how parents are affected by and adapt to incidents such as preterm birth. As shown, the mothers of hospitalized preterm infants are likely to experience clinically significant sleep disturbances in the early postpartum phase, which is surprising because many mothers slept at home and did not provide care for the preterm infants [46–51]. These findings are supported elsewhere. Blomqvist et al. [63] found significantly more severe levels of insomnia in mothers compared to fathers early postpartum, and maternal insomnia levels were independent of sleep location. A plausible explanation was that the mothers experienced the same amount of stress and anxiety regardless of sleep location [63]. Mothers of preterm infants are at risk for poor sleep, and they need help and support from health care personnel to meet their basic need for sleep, regardless of sleep location. The findings of this review not only illustrate that mothers’ sleep is challenged but also highlight the major psychological stress and emotional challenges that mothers face. In addition to being associated with poor sleep, being a mother of a hospitalized preterm infant is associated with stress, anxiety, fatigue, depression and risk of poor HRQoL [46–51, 56]. In a recent study, Busse et al. [10] reported similar findings. Parents in the NICU experienced emotional and physical constellations of anxiety, depression, fatigue and sleep disruption. The findings showed that mothers of preterm infants experience a transition to motherhood that has the potential to disrupt the entire balance of their lives, and the combination of poor sleep and emotional responses described requires more attention and future effort from healthcare personnel. In neonatal care, one of the greatest challenges facing neonatal nurses is how to provide care that supports the needs of mothers and infants. Nurses must understand maternal perceptions, expectations and needs to meet these challenges [64]. Accordingly, NICU nurses must be aware and show awareness of maternal expectations and experiences. Recognizing different types of parental reactions is essential if nurses are to optimize the outcome for the parents [65]. Suggested preventive care for postpartum women includes assessing maternal sleep and depression during the postpartum period and providing sleep hygiene information to promote sleep for NICU parents [46]. Routine screening tools and inquiries about stress and sleep patterns have also been introduced as efforts to recognize symptoms in the early postpartum phase [51]. Advice from healthcare providers to maintain consistent sleep-wake schedules, as well as instruction in relaxation techniques, can be other helpful approaches to achieving more sleep and better sleep quality [66]. During the hospitalization period, parents must be confident and prepared – with tailored information and guidance – to take their infants home [67]. Ideally, neonatal nurses could begin parental support processes while infants are still in the NICU and continue these processes after discharge [68]. In future research, efforts to promote sleep must be evaluated in studies with a long-term perspective, and interventions should be tested in studies with large study samples using assorted research instruments to assess sleep. Surprisingly, none of the studies in this review evaluated parental sleep over time by using modern research tools such as actigraphs or sleep diaries. The use of actigraphs has been shown to be a feasible way to provide sleep data in large epidemiologic studies and is considered important in follow-up studies and for examining treatment efficacy in clinical outcomes [69]. A recent study showed that actigraphs should be used for at least 7 nights to measure total sleep time, and sleep efficiency should be measured for at least 5 nights [70]. Additionally, sleep diaries have become widely used in modern sleep research and are considered today’s gold standard for subjective sleep assessment [71]. Recommendations for future research are therefore to explore sleep among parents of preterm infants with both objective and subjective research tools, evaluate sleep characteristics over time, and collect data over longer time periods. Compared to recent studies, future research could also benefit from more variation in study design in the exploration of parental sleep. Qualitative study designs are considered to be particularly well suited to understanding causal relationships [72]. Qualitative studies may provide a deeper understanding of how sleep is affected and experienced by parents and may help to explore the complex association between sleep and health. Because fathers are equal partners in care for the preterm infant [73], future research could benefit from using study populations that also include fathers. Carter et al. [74] found that infant prematurity negatively impacted fathers. Fathers of preterm infants have also reported higher stress rates compared to mothers 4 months postpartum [75], but more needs to be learned about the role of sleep.

### Strengths and limitations

To the best of the authors’ knowledge, this was the first scoping review to summarize the existing evidence on sleep and its relationships to health in parents of preterm infants. The strengths of this review are its broad and comprehensive search in the electronic databases, inclusion of quantitative and qualitative literature, and lack of restrictions on date range in the literature searches. One limitation of the findings in this scoping review is the lack of cultural and geographical breadth. The included research was predominated by studies from U.S, resulting in findings with geographical concentration in western societies, and study populations characterized by ethnic specificity [46, 47, 50, 51]. Therefore, the extent to which the results can be used and generalized to parents in other geographical areas worldwide remains unclear.

Another limitation was the lack of qualitative research in this review’s findings. Qualitative research is considered to be especially useful in contributing with in debt knowledge and a deeper understanding of complex human phenomenon’s [72], and could have given valuable knowledge on this reviews topic. The existing research was also limited in time, most of the articles focused on the two first weeks after the preterm birth. Therefore, the knowledge of what happens to parental sleep and health in the later timeframe after birth, was limited. In addition to this, substantial heterogeneity in how parental sleep was documented, both subjectively and objectively, made it difficult to compare the results in the later, delayed postpartum period. A last limitation was also the lack of knowledge on fathers’ sleep and health, since the overall research in this review was performed on mothers.

## Conclusions

This review addresses concerns about parental sleep, health and well-being. Despite limitations on the applicability of the results to parents globally, efforts to promote sleep and health may be a prominent issue for health care providers in hospital settings worldwide. More needs to be learned about fathers’ sleep and health and about whether the long-term consequences to sleep and health differ for parents of preterm infants. Knowledge of sleep and health characteristics might be the first step toward developing efforts and interventions to promote a healthy parent population.

## Abbreviations

5-HTTLPR: Serotonin-transporter-linked polymorphic region
HRQoL: Health related quality of life
MeSH: Medical subject headings
NICU: Neonatal intensive care unit
PICOC: Population, intervention/exposure, comparison, outcome, context
PRISMA: Preferred Reporting Items for Systematic Reviews and Meta-Analyses
PROM: Patient reported outcome measures
REM: Rapid eye movement sleep
R-GI: Relaxation guided imagery intervention
TST: Total sleep time
U.S: United states
WHO: World health organization

## References

[1] World health organization [WHO]. Preterm birth 2016 http://www.who.int/mediacentre/factsheets/fs363/en/. Accessed 06.11.2017.

[2] Blencowe H, Cousens S, Oestergaard MZ, Chou D, Moller AB, Narwal R (2012). National, regional, and worldwide estimates of preterm birth rates in the year 2010 with time trends since 1990 for selected countries: a systematic analysis and implications. Lancet.

[3] Miles SM, Holditch-Davis AD, Schwartz AT, Scher AM (2007). Depressive symptoms in mothers of prematurely born infants. J DevBehav Pediatr.

[4] Mousavi SS, Chaman R, Khosravi A, Mohagheghi P, Mousavi SA, Keramat A (2016). The needs of parents of preterm infants in Iran and a comparison with those in other countries: a systematic review and meta-analysis. Iran J Pediatr.

[5] Davis L, Edwards H, Mohay H, Wollin J (2003). The impact of very premature birth on the psychological health of mothers. Early Hum Dev.

[6] Singer LT, Salvator A, Guo S, Collin M, Lilien L, Baley J (1999). Maternal psychological distress and parenting stress after the birth of a very low-birth-weight infant. JAMA.

[7] Holditch-Davis D, Bartlett TR, Blickman AL, Miles MS (2003). Posttraumatic stress symptoms in mothers of premature infants. J Obstet Gynecol Neonatal Nurs.

[8] Lau R, Morse CA (2003). Stress experiences of parents with premature infants in a special care nursery. Stress Health.

[9] Edell-Gustafsson U, Angelhoff C, Johnsson E, Karlsson J, Morelius E (2015). Hindering and buffering factors for parental sleep in neonatal care. A phenomenographic study. J Clin Nurs.

[10] Busse M, Stromgren K, Thorngate L, Thomas KA (2013). Parents' responses to stress in the neonatal intensive care unit. Crit Care Nurse.

[11] Stremler R, Dhukai Z, Wong L, Parshuram C (2011). Factors influencing sleep for parents of critically ill hospitalised children: a qualitative analysis. Intensive Crit Care Nurs.

[12] Luyster Faith S. (2013). Sleep and Health. Encyclopedia of Behavioral Medicine.

[13] Lee KA, Redeker NS, Mcenany GP (2011). Sleep promotion in the childbearing family. Sleep disorders and sleep promotion in nursing practice.

[14] Hall MH. Sleep. In: Gellman MD, Turner JR, editors. Encyclopedia of behavioral medicine. New York, NY: Springer New York; 2013. p. 1795–1799.

[15] Eugene AR, Masiak J (2015). The neuroprotective aspects of sleep. MEDtube science.

[16] Landis CA, Redeker NS, McEnany GP (2011). Physiological and behavioural aspects of sleep. Sleep disorders and sleep promotion in nursing practice.

[17] Roberts RE, Duong HT (2014). The prospective association between sleep deprivation and depression among adolescents. Sleep.

[18] Zee PC, Turek FW (2006). Sleep and health - Everywhere and in both directions. Arch Intern Med.

[19] Insana SP, Montgomery-Downs HE (2013). Sleep and sleepiness among first-time postpartum parents: a field- and laboratory-based multimethod assessment. Dev Psychobiol.

[20] Romano M, Cacciatore A, Giordano R, La Rosa B (2010). Postpartum period: three distinct but continuous phases. J Prenat Med.

[21] Holditch-Davis D, Miles MS (2000). Mothers’ stories about their experiences in the neonatal intensive care unit. Neonatal netw.

[22] Lefkowitz D, Baxt C, Evans J (2010). Prevalence and correlates of posttraumatic stress and postpartum depression in parents of infants in the neonatal intensive care unit (NICU). J Clin Psychol Med Settings.

[23] Alkozei A, McMahon E, Lahav A (2014). Stress levels and depressive symptoms in NICU mothers in the early postpartum period. J Matern Fetal Neonatal Med.

[24] Shaw RJ, Deblois T, Ikuta L, Ginzburg K, Fleisher B, Koopman C (2006). Acute stress disorder among parents of infants in the neonatal intensive care nursery. Psychosomatics.

[25] Vigod SN, Villegas L, Dennis CL, Ross LE (2010). Prevalence and risk factors for postpartum depression among women with preterm and low-birth-weight infants: a systematic review. BJOG.

[26] Huang YS, Paiva T, Hsu JF, Kuo MC, Guilleminault C. Sleep and breathing in premature infants at 6 months post-natal age. BMC Pediatr 2014;14. doi: ARTN 30310.1186/s12887%2D014%2D0303-6. 10.1186/s12887-014-0303-6 PMC4272529 25510740

[27] Stangenes KM, Fevang SK, Grundt J, Donkor HM, Markestad T, Hysing M (2017). Children born extremely preterm had different sleeping habits at 11 years of age and more childhood sleep problems than term-born children. Acta Paediatr.

[28] Posmontier B (2008). Sleep quality in women with and without postpartum depression. J Obstet Gynecol Neonatal Nurs.

[29] Gay CL, Lee KA, Lee SY (2004). Sleep patterns and fatigue in new mothers and fathers. Biol Res Nurs.

[30] Goyal D, Gay CL, Lee KA (2007). Patterns of sleep disruption and depressive symptoms in new mothers. J Perinat Neonatal Nurs..

[31] Dennis CL, Ross L (2005). Relationships among infant sleep patterns, maternal fatigue, and development of depressive symptomatology. Birth.

[32] Stickland A, Clayton E, Sankey R, Hill CM (2016). A qualitative study of sleep quality in children and their resident parents when in hospital. Arch Dis Child.

[33] Medina AM, Lederhos CL, Lillis TA (2009). Sleep disruption and decline in marital satisfaction across the transition to parenthood. Fam Syst Health..

[34] Kong LP, Cui Y, Qiu YF, Han SP, Yu ZB, Guo XR (2013). Anxiety and depression in parents of sick neonates: a hospital-based study. J Clin Nurs.

[35] Clark CA, Woodward LJ, Horwood LJ, Moor S (2008). Development of emotional and behavioral regulation in children born extremely preterm and very preterm: biological and social influences. Child Dev.

[36] Treyvaud K, Anderson VA, Lee KJ, Woodward LJ, Newnham C, Inder TE (2010). Parental mental health and early social-emotional development of children born very preterm. J Pediatr Psychol.

[37] Flacking R, Lehtonen L, Thomson G, Axelin A, Ahlqvist S, Moran VH (2012). Closeness and separation in neonatal intensive care. Acta Paediatr.

[38] Giallo R, Rose N, Cooklin A, McCormack D (2013). In survival mode: mothers and fathers' experiences of fatigue in the early parenting period. J Reprod Infant Psychol.

[39] Stremler R, Wong L, Parshuram C (2008). Practices and provisions for parents sleeping overnight with a hospitalized child. J Pediatr Psychol.

[40] Arksey H, O'Malley L (2005). Scoping studies: towards a methodological framework. Int J Soc Res Methodol.

[41] Levac D, Colquhoun H, O'Brien KK (2010). Scoping studies: advancing the methodology. Implement Sci.

[42] Booth A, Sutton A, Papaioannou D. Systematic approaches to a successful literature review. 2nd ed. ed. Los Angels, Cal: Sage; 2016.

[43] Booth A (2008). Unpacking your literature search toolbox: on search styles and tactics. Health Inf Libr J.

[44] Moher D, Liberati A, Tetzlaff J, Altman DG (2009). Preferred reporting items for systematic reviews and meta-analyses: the PRISMA statement. Ann Int Med.

[45] World health organization [WHO]. About WHO. 2017. http://www.who.int/about/mission/en/. Accessed 06.11.2017.

[46] Lee SY, Aycock DM, Moloney MF (2013). Bright light therapy to promote sleep in mothers of low-birth-weight infants: a pilot study. Biol Res Nurs..

[47] Lee SY, Grantham CH, Shelton S, Meaney-Delman D (2012). Does activity matter: an exploratory study among mothers with preterm infants?. Arch Womens Ment Health.

[48] Lee SY, Hsu HC (2012). Stress and health-related well-being among mothers with a low birth weight infant: the role of sleep. Soc Sci Med.

[49] Lee SY, Kimble LP (2009). Impaired sleep and well-being in mothers with low-birth-weight infants. J Obstet Gynecol Neonatal Nurs.

[50] Lee Shih-Yu, Hsu Hui-Chin (2016). Genetic Susceptibility and Sleep Disturbance in Black Mothers of Preterm Infants: An Exploratory Study. SAGE Open Nursing.

[51] Shelton SL, Meaney-Delman DM, Hunter M, Lee S-Y. Depressive symptoms and the relationship of stress, sleep, and well-being among NICU mothers. J Nurs Educ Pract. 2014;4. 10.5430/jnep.v4n8p70.

[52] Williams PD, Williams AR (1997). Transition from hospital to home by mothers of preterm infants: path analysis results over three time periods. Fam Syst Health.

[53] Gennaro S, Fehder W (2000). Health behaviors in postpartum women. Fam Commun Health.

[54] McMillen IC, Mulvogue HM, Kok JSM, Deayton JM, Nowak R, Adamson TM (1993). Circadian -rhythms in sleep and wakefulness in salivary melatonin and cortisol concentrations in mothers of term and preterm infants. Sleep.

[55] Wollenhaupt JM (2010). The experience of mothers and fathers with their premature infant in the family home.

[56] Schaffer L. The impact of guided imagery on sleep quality in mothers of preterm infants. California: University of San Diego; 2012.

[57] Schaffer L, Jallo N, Howland L, James K, Glaser D, Arnell K (2013). Guided imagery: an innovative approach to improving maternal sleep quality. J Perinat Neonatal Nurs.

[58] Martin JL, Hakim AD (2011). Wrist Actigraphy. Chest.

[59] Nelson EC, Eftimovska E, Lind C, Hager A, Wasson JH, Lindblad S (2015). Patient reported outcome measures in practice. BMJ.

[60] Ong JC, Arnedt T, Gehrman PR, Kryger R, Dement WC (2017). Insomnia diagnosis, assessment and evaluation. Principles and practice of sleep medicine.

[61] Stefana A, Lavelli M (2017). Parental engagement and early interactions with preterm infants during the stay in the neonatal intensive care unit: protocol of a mixed-method and longitudinal study. BMJ Open.

[62] Feldman R (2007). Parent-infant synchrony and the construction of shared timing; physiological precursors, developmental outcomes, and risk conditions. J Child Psychol Psychiatry.

[63] Blomqvist YT, Nyqvist KH, Rubertsson C, Funkquist EL (2017). Parents need support to find ways to optimise their own sleep without seeing their preterm infant's sleeping patterns as a problem. Acta Paediatr.

[64] Hurst I (2001). Mothers' strategies to meet their needs in the newborn intensive care nursery. J Perinat Neonatal Nurs..

[65] Heidari H, Hasanpour M, Fooladi M (2013). The experiences of parents with infants in neonatal intensive care unit. Iran J Nurs Midwifery Res.

[66] Stremler R, Dhukai Z, Pullenayegum E, Weston J, Wong L, Parshuram C (2014). Sleep, sleepiness, and fatigue outcomes for parents of critically ill children. Pediatr Crit Care Med.

[67] Aagaard H, Uhrenfeldt L, Ludvigsen M, Fegran L (2015). Parents’ experiences of transition when their infants are discharged from the neonatal intensive care unit: a systematic review protocol. JBI Database System Rev Implement Rep.

[68] Adama EA, Bayes S, Sundin D (2016). Parents’ experiences of caring for preterm infants after discharge from neonatal intensive care unit: a meta-synthesis of the literature. J Neonatal Nurs.

[69] Stone KL, In A-ISA, Kryger R, Dement WC (2017). Principles and practice of sleep medicine.

[70] Aili K, Astrom-Paulsson S, Stoetzer U, Svartengren M, Hillert L (2017). Reliability of Actigraphy and subjective sleep measurements in adults: the Design of Sleep Assessments. J Clin Sleep Med.

[71] Short MA, Arora T, Gradisar M, Taheri S, Carskadon MA (2017). How many sleep diary entries are needed to reliably estimate adolescent sleep?. Sleep.

[72] Polit DF, Beck CT (2010). Essentials of nursing research : appraising evidence for nursing practice.

[73] Shields L, Zhou H, Pratt J, Taylor M, Hunter J, Pascoe E (2012). Family-centred care for hospitalised children aged 0-12 years. Cochrane Database Syst Rev.

[74] Carter JD, Mulder RT, Bartram AF, Darlow BA (2005). Infants in a neonatal intensive care unit: parental response. Arch Dis Child Fetal Neonatal Ed.

[75] Shaw RJ, Bernard RS, Deblois T, Ikuta LM, Ginzburg K, Koopman C (2009). The relationship between acute stress disorder and posttraumatic stress disorder in the neonatal intensive care unit. Psychosomatics.

